# Fluid balance and urine volume are independent predictors of mortality in acute kidney injury

**DOI:** 10.1186/cc12484

**Published:** 2013-01-24

**Authors:** Catarina Teixeira, Francesco Garzotto, Pasquale Piccinni, Nicola Brienza, Michele Iannuzzi, Silvia Gramaticopolo, Francesco Forfori, Paolo Pelaia, Monica Rocco, Claudio Ronco, Clara Belluomo Anello, Tiziana Bove, Mauro Carlini, Vincenzo Michetti, Dinna N Cruz

**Affiliations:** 1 International Renal Research Institute Vicenza (IRRIV), Viale Rodolfi 37, Vicenza, 36100, Italy; 2 Department of Nephrology, Dialysis and Transplantation, St. Bortolo Hospital, Viale Rodolfi 37, Vicenza, 36100, Italy; 3 Department of Intensive Care and Anesthesiology, St. Bortolo Hospital, Viale Rodolfi 37, Vicenza, 36100, Italy; 4 Department of Anesthesia and Intensive Care, Emergency and Organ Transplantation - University of Bari, Pizza G. Cesare 11, Bari, 70124, Italy; 5 Intensive Care Unit, Department of Anesthesia and Resuscitation, Federico II University Hospital, Via S. Pansini 5, Naples, 80131, Italy; 6 Division of Anesthesiology and Intensive Care, Department of Surgery, University of Pisa, Via Roma 67, Pisa, 56100, Italy; 7 Intensive Care Unit, University Hospital Umberto I - G.M. Lancisi - G. Salesi, Via Tronto 10/A, Torrette di Ancona, 60020, Italy; 8 Department of Anesthesiology and Intensive Care, University of Rome "La Sapienza", Viale del Policlinico 155, Rome, 00161, Italy; 9 Intensive Care Unit, Emergency Department of Anesthesiological and Surgical Science 2 (SUN), University Hospital, Via Santa Maria di Costantinopoli 104, Naples, 80138, Italy; 10 Department of Cardiothoracic Anesthesia and Intensive Care, Vita-Salute San Raffaele University, San Raffaele Scientific Institute, Via Olgettina 60, Milan, 20132, Italy; 11 Department of Anesthesia and Intensive Care, A.O.U.I. University of Verona, Piazzale Scuro, Verona, 37134, Italy; 12 Department of Intensive Care and Anesthesiology - Catholic University of Sacred Heart, Largo A. Gemelli 8, Rome, 00168, Italy

## Abstract

**Introduction:**

In ICUs, both fluid overload and oliguria are common complications associated with increased mortality among critically ill patients, particularly in acute kidney injury (AKI). Although fluid overload is an expected complication of oliguria, it remains unclear whether their effects on mortality are independent of each other. The aim of this study is to evaluate the impact of both fluid balance and urine volume on outcomes and determine whether they behave as independent predictors of mortality in adult ICU patients with AKI.

**Methods:**

We performed a secondary analysis of data from a multicenter, prospective cohort study in 10 Italian ICUs. AKI was defined by renal sequential organ failure assessment (SOFA) score (creatinine >3.5 mg/dL or urine output (UO) <500 mL/d). Oliguria was defined as a UO <500 mL/d. Mean fluid balance (MFB) and mean urine volume (MUV) were calculated as the arithmetic mean of all daily values. Use of diuretics was noted daily. To assess the impact of MFB and MUV on mortality of AKI patients, multivariate analysis was performed by Cox regression.

**Results:**

Of the 601 included patients, 132 had AKI during their ICU stay and the mortality in this group was 50%. Non-surviving AKI patients had higher MFB (1.31 ± 1.24 versus 0.17 ± 0.72 L/day; *P *<0.001) and lower MUV (1.28 ± 0.90 versus 2.35 ± 0.98 L/day; *P *<0.001) as compared to survivors. In the multivariate analysis, MFB (adjusted hazard ratio (HR) 1.67 per L/day, 95%CI 1.33 to 2.09; <0.001) and MUV (adjusted HR 0.47 per L/day, 95%CI 0.33 to 0.67; <0.001) remained independent risk factors for 28-day mortality after adjustment for age, gender, diabetes, hypertension, diuretic use, non-renal SOFA and sepsis. Diuretic use was associated with better survival in this population (adjusted HR 0.25, 95%CI 0.12 to 0.52; <0.001).

**Conclusions:**

In this multicenter ICU study, a higher fluid balance and a lower urine volume were both important factors associated with 28-day mortality of AKI patients.

## Introduction

Early and appropriate goal-directed fluid therapy is fundamental in acute resuscitation of critically ill patients [1,2]; however, it is almost always associated with a certain degree of fluid overload (FO) [3,4], which promotes tissue edema that could potentially contribute to progressive organ dysfunction [5,6].

There is a growing amount of evidence supporting the relation between FO and unfavorable outcomes in critically ill patients. This has been demonstrated in general ICUs [7] and in specific clinical settings, such as acute lung injury/acute respiratory distress syndrome (ALI/ARDS) [8,9], in patients undergoing abdominal surgery [10,11], cardiac surgery [12] and in septic patients [13]. With regard to acute kidney injury (AKI) in particular, multiple pediatric studies have shown that a greater FO at the time of renal replacement therapy (RRT) is associated with higher mortality [14-16]. In a small study of 81 adult AKI patients who underwent RRT, a volume-related weight gain (VRWG) >10%, used as a surrogate for FO, was similarly associated with poor outcomes [17]. An association between FO and higher mortality has also been shown in critically ill patients with less severe forms of AKI [18-21]. In this context, FO has emerged as a potential 'biomarker' in critical illness, and it has been proposed that its prevention may be an important determinant of survival, particularly in AKI [3].

Oliguria is a common situation in the ICU and is considered an early and sensitive biomarker of renal injury because it may precede a creatinine-based diagnosis of AKI [22,23]. Furthermore, in multicenter studies on critically ill patients with AKI, the presence of oliguria has been associated with higher mortality [20,24-26]. Indeed, the evidence points out that both FO and oliguria are important predictors of mortality in critically ill patients. Fluid accumulation is a logical and expected complication of oliguric AKI due to impaired water and sodium excretion [20,27]. It remains unclear whether their effects are independent of each other or if FO is merely in the causal pathway between oliguria and death. Interestingly, very few studies have adjusted for urine output in the analysis on FO and mortality. In 81 AKI patients requiring RRT, both oliguria and FO were independent predictors of mortality [17] and in a *post hoc *analysis of 306 AKI patients from the Fluid and Catheter Treatment Trial (FACTT), FO was shown to be independently associated with mortality both in oliguric and non-oliguric patients [19]. In a large multicenter European study [20] on critically ill patients with AKI, a positive fluid balance was an important factor associated with increased 60-day mortality, but there was no adjustment for urine volume or oliguria.

The aim of the present study is to evaluate the impact of both fluid balance and urine volume on outcomes and determine whether they behave as independent predictors of mortality in adult ICU patients with AKI.

## Materials and methods

### Study design and data collection

We performed a secondary analysis of data from 601 patients enrolled in a prospective multicenter ICU cohort study of adult patients (age ≥18 years) admitted to 10 ICUs from September 2009 to April 2010 (NEFROlogia e Cura INTensiva (NEFROINT) Study), designed to describe the epidemiology of AKI in Italian ICUs [28]. Detailed methods of data collection have been previously described [29]. Collected data included demographics, anthropometrics, admission diagnosis, comorbidities, daily vital signs and laboratory data to calculate automatically the Acute Physiology and Chronic Health Evaluation II (APACHE II) [30], the Simplified Acute Physiology Score II (SAPS II) [31] and the Sequential Organ Failure Assessment (SOFA) [32] scores. RRT details and mortality were also reported. Furthermore, 24-hour urine output, worst 6-hour and worst 12-hour urine output, fluid balance and use of diuretics were recorded daily. Furosemide was the only diuretic used in all the centers. Fluid balance was calculated daily as the difference between fluid intake and fluid output. Fluid output included all body fluids, including urine and, if applicable, dialysis ultrafiltrate. This study was approved by the ethics committee of St. Bortolo Hospital, Vicenza, Italy. Because of the anonymous and non-interventional nature of the study, the ethics committees of the participating study centers (see list below) waived the need for informed consent.

### Definitions

AKI was defined according to the renal SOFA score as a creatinine >3.5 mg/dL or a urine output <500 mL/day, as used by the Sepsis Occurrence in Acutely Ill Patients (SOAP) study investigators [20]. Patients who received RRT were assigned a renal SOFA score of 4, regardless of the serum creatinine or urine output. AKI was considered 'early' if it occurred in the first two days of ICU admission and 'late' if it occurred more than two days after ICU admission [20]. Oliguria was defined as a urine output of less than 500 mL/day. Mean fluid balance (MFB) and mean urine volume (MUV) were calculated as the arithmetic mean of the daily values during the patient's ICU stay. The SOFA score was expressed in two ways: total score and the score without the renal component (non-renal SOFA). For sensitivity analysis, we also defined AKI using the Risk, Injury, Failure, Loss of kidney function and End-stage kidney disease (RIFLE) consensus [33].

### Statistical analysis

Continuous variables were expressed as both mean ± SD and as median (IQR) and compared between any two groups (for example, AKI versus non-AKI, survivors versus non-survivors, early versus late AKI and so on), using the t-test or Mann-Whitney U test, as appropriate. Normality of distribution was evaluated by visual inspection of histograms and by the Kolmogorov Smirnov test. Categorical variables are expressed as number of cases and proportion and compared with the Mantel-Haenszel Chi-square test or the Fisher exact test.

To evaluate the influence of MFB and MUV on the survival time of AKI patients, exploratory univariate analysis for several variables was first performed to identify possible confounders associated with 28-day mortality. A multivariable Cox regression analysis was then performed using a forward stepwise selection method, criteria for entry 0.05 and for removal 0.10. The assumption of proportional hazards was checked graphically using log(-log(survival probability)) plots and was found appropriate. Variables considered for multivariable analysis were age, gender, co-morbid disease, sepsis, non-renal SOFA, and MFB, as were used in a previous study [20], with the addition of MUV and diuretic use. Age, sepsis and diuretic use were all significant on univariate analysis. APACHE II, SAPS II, and total and non-renal SOFA scores were also significant on univariate analysis, but the strongest association with mortality was seen with non-renal SOFA, and this was used in all multivariable models. We tested for collinearity among all variables. From the Cox model, hazard ratio (HR) and 95% CIs were generated. We also performed a number of sensitivity analyses. First, since MFB and MUV were found to be collinear, we repeated the analysis using an interaction term MFB*MUV. Second, we also expressed urine output as the presence of oliguria anytime during the ICU stay and the duration of oliguria (defined as the proportion of ICU days complicated by oliguria) as an alternate representation of urine volume in the models. Third, diuretic use was also expressed as duration of diuretic use (defined by the proportion of ICU days in which diuretics were used). The 28-day survival by timing of AKI, oliguria and diuretic use were additionally evaluated graphically using the Kaplan-Meier product limit survival plot. All statistical analysis was performed using the SPSS 19.0 (SPSS Inc, Chicago, IL, USA) software package, with a two-sided *P *value <0.05 considered as statistically significant.

## Results

### Study population

Of the 601 patients enrolled in the NEFROINT study [28], 25 were excluded because of the presence of end-stage renal disease on chronic RRT and three other patients were excluded because of incomplete data on fluid balance and urine output, leaving 573 patients for analysis. Characteristics of the entire cohort are shown in Additional file 1, Table S1. The mean age was 63.0 ± 17.3 years and 59.5% were men. All-cause 28-day mortality was 21.8% and mean ICU length of stay was 10.1 ± 12.0 days. Of these patients, 132 (23%) developed AKI.

### Acute kidney injury

AKI patients were older, more likely to be diabetic and to have sepsis or a cardiovascular diagnosis at ICU admission and higher illness severity scores than non-AKI [see Additional file 1, Table S1]. MFB was higher (0.7 ± 1.2 versus 0.5 ± 1.1 L/day; *P *= 0.008) and MUV was lower (1.8 ± 1.1 versus 2.6 ± 1.3 L/day; *P *<0.001) in the AKI group. Diuretics were used more commonly on AKI patients and for a larger proportion of ICU days (Table 1). Mortality was higher among AKI patients (50% versus 13.4%; *P *<0.001) and ICU stay was longer.

**Table 1:** Fluid balance, urine volume and diuretic use by presence or absence of AKI.

	All (number = 573)	AKI (number = 132)	Non-AKI (number = 441)	*P*
Mean fluid balance/24hours (L)	0.52 ± 1.11 0.40 (0.04 to 0.97)	0.74 ± 1.16 0.48 (0.02 to 1.26)	0.45 ± 1.09 0.38 (-0.05 to 0.86)	0.008
Mean urine volume/24hours (L)	2.43 ± 1.31 2.39 (1.66 to 3.06)	1.81 ± 1.08 1.88 (0.87 to 2.73)	2.62 ± 1.32 2.54 (1.86 to 3.11)	<0.001
Use of diuretics (%)	365 (63.7%)	102 (77.3%)	263 (59.6%)	<0.001
Duration of diuretic use (days)	5.3 ± 8.5 2 (0 to7)	8.3 ± 11.3 4 (1 to 10)	4.4 ± 7.2 2 (0 to 6)	<0.001
% ICU days with diuretics	43.2 ± 39.6% 42.9 (0 to 80.5%)	51.0 ± 37.6% 51.9 (8.7 to 87.5%)	(40.9 ± 39.9%) 38.5 (0 to 78.4%)	0.010

AKI, acute kidney injury.

AKI patient characteristics according to outcome are shown in Table 2. Non-surviving AKI patients were older, had higher illness severity scores and were more likely to be septic. The MFB was higher (1.3 ± 1.2 versus 0.2 ± 0.7 L/day; *P *<0.001) and the MUV was lower (1.3 ± 0.9 versus 2.3 ± 1.0 L/day; *P *<0.001) among non-survivors. Oliguria was also more common in non-survivors (83.3% versus 54.5%; *P *= 0.001) and persisted for a larger proportion of the ICU stay (35.5 ± 34.5% versus 13.6 ± 21.9%; *P *<0.001).

**Table 2:** AKI patient characteristics by outcome.

	All AKI (number = 132)	Survivors (number = 66)	Non-Survivors (number = 66)	*P*
Male gender	84 (63.6%)	43 (34.8%)	41 (62.1%)	0.86
Age (years)	66.3 ± 14.1 68.5 (38 to 57.5)	63.1 ± 15.6 64 (52.8 to 76)	69.5 ± 11.7 74 (61 to 78.3)	0.015
**ICU Admission**				
SAPS II	50.0 ± 14.5 48 (38 to 57.5)	46.3 ± 13.5 42 (37 to 52.8)	53.7 ±14.6 51.5 (43.8 to 61.3)	0.001
SOFA	8.0 ± 4.1 7 (5 to 11)	6.8 ± 3.6 7 (4 to 9.3)	9.2 ± 4.3 8.5 (6 to 12)	0.002
Non-renal SOFA	6.6 ± 3.5 6 (4 to 9)	5.4 ± 2.9 5 (3 to 7)	7.8 ± 3.6 7 (5 to 11)	<0.001
APACHE II	22.3 ± 8.4 21 (16 to 28)	20.3 ± 7.7 19 (15 to 25.3)	24.4 ± 8.6 24 (16.8 to 31)	0.006
Vasoactive therapy	56 (42.4%)	21 (31.8%)	35 (53.0%)	0.022
Mechanical ventilation	96 (72.7%)	44 (66.7%)	52 (78.8%)	0.17
Serum creatinine (mg/dL)	2.3 ± 2.0 1.45 (0.9 to 3.2)	2.3 ± 2.0 1.4 (0.9 to 3.1)	2.4 ± 2.1 1.5 (0.8 to 3.3)	0.99
**Comorbid diseases**				
Diabetes	41 (31.1%)	20 (30.3%)	21 (31.8%)	1
Cardiovascular	66 (50.0%)	31 (47.0%)	35 (53.0%)	0.60
Hypertension	67 (50.8%)	29 (43.9%)	38 (57.6%)	0.16
Chronic kidney disease	19 (14.4%)	10 (15.2%)	9 (13.6%)	1
Metastatic carcinoma	4 (3.0%)	3 (4.5%)	1 (1.5%)	0.62
Hematologic malignancy	6 (4.5%)	2 (3.0%)	4 (6.1%)	0.68
**Category of ICU admission diagnosis**				
Respiratory	44 (33.3%)	20 (30.3%)	24 (36.4%)	0.58
Neurologic	15 (11.4%)	9 (13.6%)	6 (9.1%)	0.59
Trauma	8 (6.1%)	5 (7.6%)	3 (4.5%)	0.72
Cardiovascular	25 (18.9%)	12 (18.2%)	13 (19.7%)	1
Sepsis	15 (11.4%)	6 (9.1%)	9 (13.6%)	0.59
**ICU course**				
Sepsis	61 (46.2%)	23 (34.8%)	38 (57.6%)	0.014
Early AKI (%)	83 (62.9%)	39 (59.1%)	44 (66.7%)	0.47
Mean fluid balance/24hours (L)	0.74 ± 1.16 0.48 (0.02 to 1.26)	0.17 ± 0.72 0.02 (-0.17 to 0.48)	1.31 ± 1.24 1.09 (0.49 to 1.85)	<0.001
Oliguria	91 (68.9%)	36 (54.5%)	55 (83.3%)	0.001
Duration of oliguria (days)	2.8 ± 5.9 1 (0 to 3)	1.6 ± 4.0 1 (0 to 1)	4.0 ± 7.2 2 (1 to 4)	<0.001
% of ICU days with oliguria	24.6 ± 30.8% 11.9% (0 to 37.1%)	13.6 ± 21.9% 2.8% (0 to 20.7%)	35.5 ± 34.5 25% (6.7 to 62.0%)	<0.001
Mean urine volume/24hour(L)	1.81 ± 1.08 1.88 (0.87 to 2.73)	2.35 ± 0.98 2.49 (1.61 to 3.12)	1.28 ± 0.90 1.22 (0.55 to 1.96)	<0.001
Use of diuretics (%)	102 (77.3%)	55 (83.3%)	47 (71.2%)	0.15
Duration of diuretic use (days)	8.3 ± 11.3 4 (1 to 10.8)	10.3 ± 13.5 4.5 (1 to 13)	6.4 ± 8.3 3 (0 to 10)	0.11
% of ICU days receiving diuretics	51.0 ± 37.6 51.9% (8.7 to 87.5%)	56.2 ± 36.7% 55.6% (20 to 93.0%)	45.9 ± 38.1% 46.9% (0 to 82.6%)	0.11
**Outcomes**				
RRT	67 (50.8%)	31 (47.0%)	36 (54.5%)	0.49
Duration of RRT (days)(% of ICU days)	3.2 ± 6.9 1 (1 to 3)	2.2 ± 4.3 0 (0 to 3)	4.1 ± 8.7 1.5 (0 to 5)	0.14
% of ICU days on RRT	23.3 ± 31.3% 3.9% (0 to 45.2%)	15.4 ± 24.1% 0% (0 to 21.6%)	31.1 ± 35.6% 6.7% (0 to 66.7%)	0.03
RRT free days	12.1 ± 13.9 8 (3 to 16.8)	15.1 ±17.1 9 (3 to 23.3)	9.1 ± 8.8 6.5 (2.8 to 13)	0.06
Length of ICU stay (days)	15.3 ± 16.3 10 (5 to 20)	17.4 ± 18.7 11.5 (5 to 24)	13.2 ±13.2 9 (4 to 19.3)	0.45

AKI, acute kidney injury; SAPS II, Simplified Acute Physiology Score II; SOFA, Sequential Organ Failure Assessment; APACHE II, Acute Physiology and Chronic Health Evaluation II; RRT, renal replacement therapy.

Early AKI was present in 83 patients (62.9% of the AKI group) and was associated with higher illness severity scores, lower MUV (1.6 ± 1.1 versus 2.2 ± 0.9 L/day; *P *<0.001), a larger proportion of days of oliguria (32.3% versus 11.5%, *P *<0.001) and fewer RRT-free days, but a shorter ICU stay than late AKI [see Additional file 1, Table S2]. The MFB, the proportion of days with diuretics and the mortality were not different between the groups.

As expected, oliguric patients had a higher MFB (0.9 ± 1.3 versus 0.4 ± 1.0 L/day; *P *<0.001), tended to receive diuretics more often (73.6% versus 62.7%; *P *= 0.06), had higher mortality (60.4% versus 14.4%; *P *<0.001) and longer ICU stay (15.5 ± 15.8 versus 9.3 ± 10.9; *P *<0.001) than non-oliguric patients.

### Mean fluid balance, mean urine volume and mortality

**Figure 1:**
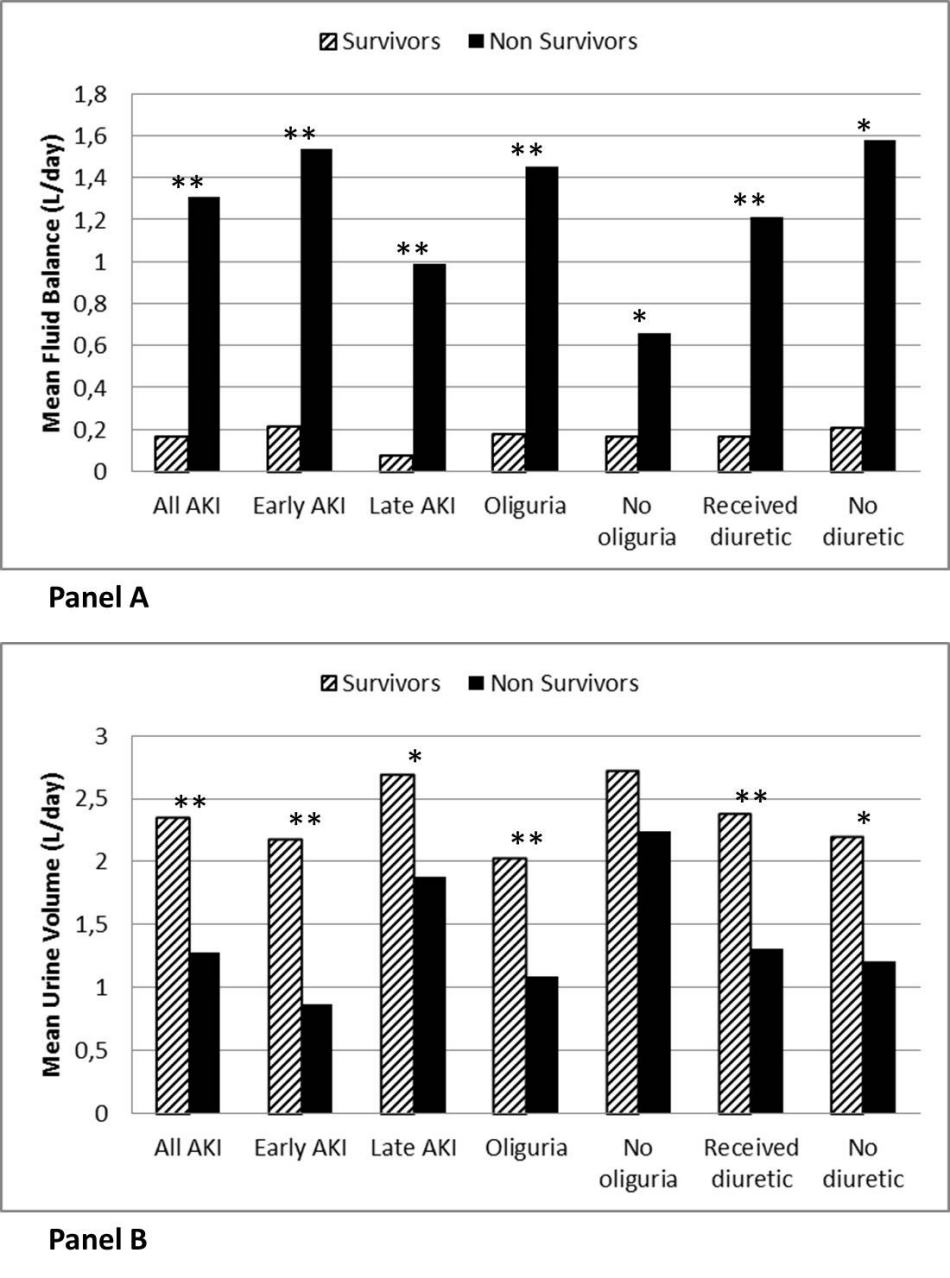
**Mean fluid balance (A) and mean urine volume (B) in survivors and non-survivors**. *P <0.05; **P <0.001. AKI, acute kidney injury.

**Figure 2:**
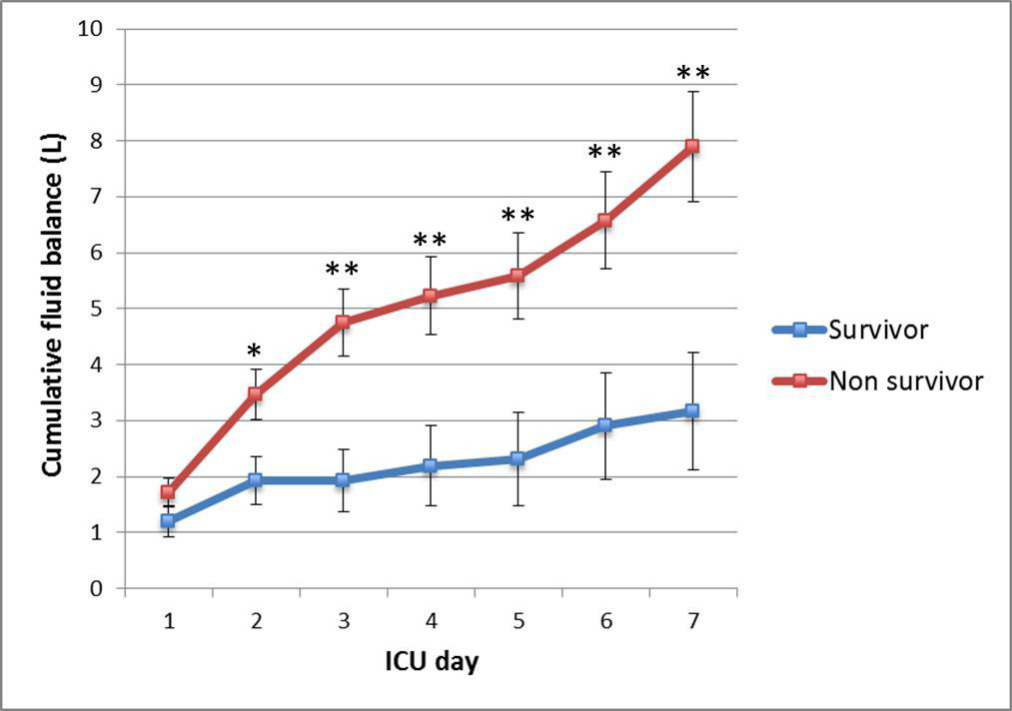
**Cumulative fluid balance in survivors and non-survivors in the first seven days of ICU stay (mean ± SEM)**. *P =0.015; **P <0.01. SEM, standard error of the mean.

In general, non-survivor AKI patients had significantly higher MFB compared to survivors (Figure 1). When analyzing the subgroups separately by AKI timing, the presence of oliguria and the use of diuretics, the same results were observed. The MUV was significantly lower among non-survivors overall, and in most of the subgroups, except in non-oliguric patients. Figure 2 demonstrates the pattern of cumulative fluid balance among survivors and non-survivors in the first seven days in the ICU. Non-survivors consistently had higher mean cumulative fluid balance throughout the ICU stay and the magnitude of the difference between the groups increased with each additional day.

**Figure 3:**
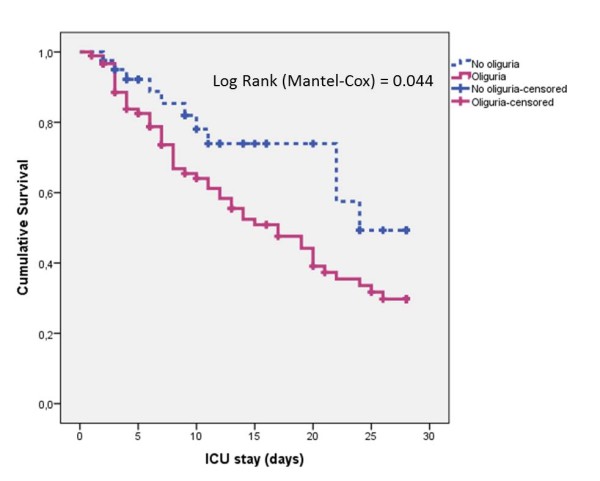
**Survival curve for 28-day mortality by the presence or absence of oliguria in the ICU**.

**Figure 4:**
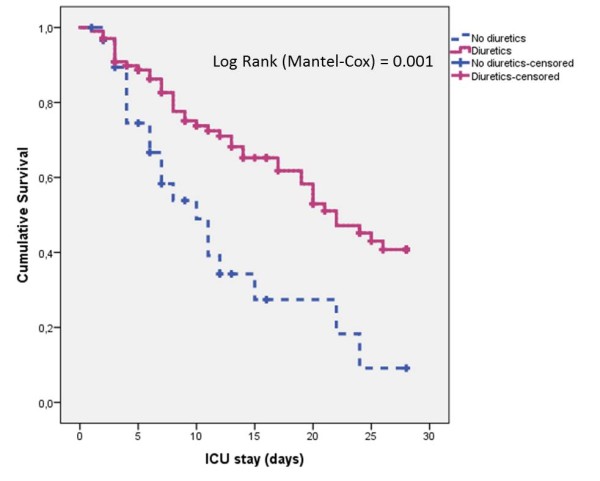
**Survival curve for 28-day mortality by the use or non-use of diuretics in the ICU**.

The 28-day survival was significantly better among non-oliguric patients (Figure 3; *P *<0.044) and in patients treated with diuretics (Figure 4; *P *= 0.001). The 28-day survival was similar between early and late AKI (not shown, *P *= 0.16 on log rank test).

On multivariate Cox regression analysis (Table 3), both MFB and MUV were independent predictors of 28-day mortality (MFB, adjusted HR 1.67, 95%CI 1.33 to 2.09 per L/day; *P *<0.001 and MUV, adjusted HR 0.47, 95%CI 0.33 to 0.67 per L/day; *P *<0.001, Model 1). Interestingly, the use of diuretics appeared to be associated with a lower risk of death in both unadjusted and adjusted analysis (adjusted HR 0.25, 95%CI 0.12 to 0.52; *P *<0.001). Collinearity was observed between MFB and MUV, and we performed a second model including the interaction between the two (MFB*MUV). In this model, the interaction term was significant; the effect of MFB was somewhat attenuated, while the effect of MUV was accentuated. Of interest, higher non-renal SOFA scores were significantly associated with increased mortality on univariate analysis (HR 1.18, 95CI% 1.10 to 1.27; *P *<0.001), but this was no longer significant on multivariable analysis (HR 1.04, 95CI% 0.96 to 1.14; *P *= 0.35).

**Table 3:** Cox regression analysis for 28-day mortality.

	Unadjusted		Adjusted			
			Model 1		Model 2	
	
	HR (95% CI)	*P*	HR (95% CI)	*P*	HR (95% CI)	*P*
Mean fluid balance (L/day)	1.99 (1.68 to 2.36)	<0.001	1.67 (1.33 to 2.09)	<0.001	1.33 (1.002 to 1.77)	0.0048
Mean urine volume (L/day)	0.38 (0.29 to 0.51)	<0.001	0.47 (0.33 to 0.67)	<0.001	0.33 (0.21 to 0.52)	<0.001
Diuretic use	0.40 (0.23 to 0.70)	0.001	0.25 (0.12 to 0.52)	<0.001	0.26 (0.13 to 0.53)	<0.001
Diabetes	1.16 (0.68 to 1.98)	0.58	2.16 (1.09 to 4.26)	0.027	2.08 (1.06 to 4.08)	0.033
Hypertension	1.76 (1.06 to 2.94)	0.03	2.18 (1.12 to 4.22)	0.021	2.17 (1.12 to 4.24)	0.023
MFB*MUV	-	-	-	-	1.53 (1.16-2.01)	0.003

Variables considered for model 1 included age, gender, co-morbid diseases (diabetes, hypertension and cardiovascular disease), non-renal SOFA, sepsis, mean fluid balance, mean urine volume and diuretic use. In model 2, MFB*MUV (interaction term between MFB and MUV) was also included. Only significant variables are shown in the table. HR, hazard ratio; MFB, mean fluid balance; MUV, mean urine volume.

In terms of sensitivity analyses [see Additional file 1, Table S3], qualitatively similar results were seen when MUV was alternatively expressed as presence of oliguria (adjusted HR 1.86, 95%CI 1.50 to 2.32 for MFB, and adjusted HR 1.35, 95%CI 0.67 to 2.71 for oliguria) or duration of oliguria (adjusted HR 1.72, 95%CI 1.37 to 2.15 for MFB, and adjusted HR 1.01, 95%CI 1.003 to 1.02 for %ICU days with oliguria), and when diuretic usage was expressed as duration of diuretics (adjusted HR 1.73, 95%CI 1.38 to 2.18 for MFB and adjusted HR 0.99, 95%CI 0.98 to 0.99 for %ICU days on diuretics). Furthermore, results were also similar when AKI was defined using RIFLE criteria (*n *= 378 AKI patients; crude mortality 28.8%). The adjusted HR for MFB was 1.51, 95%CI 1.31 to 1.74 L/day; *P *<0.001 and for MUV was 0.48, 95%CI 0.38 to 0.62 L/day; *P *<0.001 in this sensitivity analysis (Table 4).

**Table 4:** Sensitivity analysis considering presence of AKI according to RIFLE classification.

	Unadjusted		Adjusted	
	
	HR (95% CI)	*P*	HR (95% CI)	*P*
Mean fluid balance/24hours (L)	1.74 (1.56 to 1.95)	<0.001	1.51 (1.31 to 1.74)	<0.001
Mean urine volume/24hours (L)	0.39 (0.32 to 0.48)	<0.001	0.48 (0.38 to 0.62)	<0.001
Diuretic use	0.34 (0.22 to 0.52)	<0.001	0.31 (0.19 to 0.51)	<0.001
Diabetes	1.36 (0.88 to 2.10)	0.17	1.75 (1.08 to 2.85)	0.023

Variables considered for the model included age, gender, co-morbid diseases (diabetes, hypertension and cardiovascular disease), non-renal SOFA, sepsis, mean fluid balance, mean urine volume and diuretic use. Only significant variables are shown in the table. AKI, acute kidney injury; HR, hazard ratio; RIFLE, Risk of renal dysfunction, Injury to the kidney, Failure of kidney function, Loss of kidney function and End-stage kidney disease.

## Discussion

Using data from a multicenter ICU cohort [28], we examined the influence of fluid balance and urine volume on outcomes among critically ill patients with AKI. The key findings of this study are: first, AKI patients had higher MFB, lower MUV, and a higher mortality than non-AKI patients; second, MFB was significantly higher in non-survivors in all AKI patients, and consistently higher across various subgroups (early and late AKI, oliguric and non-oliguric, use and non-use of diuretics, Figure 1); third, MUV was lower in non-survivors in all subgroups, except non-oliguric patients; finally, both MFB and MUV were significantly associated with increased 28-day mortality in AKI patients, and their interaction was significant as well.

Our results are consistent with prior studies on critically ill patients, both without and with AKI. In non-AKI studies, a positive fluid balance was strongly associated with increased mortality and other unfavorable outcomes in some subgroups of ICU patients, including worse lung function, longer duration of mechanical ventilation, increased post-operative complications and longer ICU stay [7-13]. Among adult AKI patients in particular, Van Biesen *et al. *[21] showed that additional fluid loading not only failed to improve renal function, but was also associated with worsening of respiratory function. The Program to Improve Care in Acute Renal Disease (PICARD) group [18] concluded that fluid weight gain >10% in AKI patients was associated with higher mortality and that the increase in mortality rate was proportional to the degree of fluid accumulation. In a study with 1,120 ICU patients with AKI, the authors showed that the MFB was an independent predictor of mortality on multivariate analysis (adjusted HR 1.21, 95%CI 1.13 to 1.28; *P *<0.001) [20]. However, these three studies did not adjust for urine volume in their models.

An important finding in the present study is that fluid balance and urine volume were both strong and significant predictors of mortality, even accounting for their interaction. We reproduced the analysis performed in AKI patients from a multicenter European ICU cohort [20] and extended it by adding the variable urine volume to the analysis. Our findings confirmed the association of both FO (adjusted HR per L/day of MFB 1.67, 95%CI 1.33 to 2.09; *P *<0.001) and reduced urine volume (adjusted HR per L/day of MUV 0.47, 95%CI 0.33 to 0.67; *P *<0.001) with adverse outcomes. Qualitatively similar findings were obtained even when the urine volume was expressed as the presence of oliguria or duration of oliguria, implying that FO is not merely an intermediate in the pathway between lower urine output and mortality. This relation is not well-explored in the literature. To our knowledge, only two adult AKI studies have adjusted for urine output in their analysis of FO and outcomes. In a cohort of 81 adult critically ill patients with severe AKI requiring RRT, the authors examined the effect of oliguria, VRWG used as a measure of FO, sepsis and APACHE II score in the multivariate model [17]. Both oliguria and VRWG ≥10% were predictors of 30-day mortality (adjusted odds ratio (OR) 3.04, 95%CI 1.10 to 8.36 and adjusted OR 2.71, 95%CI 1.05 to 6.99, respectively). In the aforementioned FACTT study, the association between fluid balance and mortality was significant in patients both with oliguria (HR per L/day of MFB 1.77, 95%CI 1.27 to 2.45) and without oliguria (HR per L/day of MFB 1.61, 95%CI 1.27 to 2.45) during the initial seven days after randomization [19]. Our findings are consistent with these studies. Furthermore, we observed that the interaction between fluid balance and urine volume was statistically significant, and still MFB and MUV individually remained predictors of 28-day mortality.

The association between oliguria and worse outcomes in AKI patients is well recognized [4,22]. In our study, the survival of non-oliguric patients was significantly better when compared to oliguric patients (Figure 3), but in both groups non-survivors had significantly higher MFB (Figure 1). Similarly to other authors [20,25], we did not find significant differences in mortality between patients who developed early or late AKI (not shown, *P *= 0.16 on log rank test) but again, non-survivors in both groups presented significantly higher MFB and lower MUV.

An interesting finding in the present study is that the use of diuretics was inversely associated with mortality, and this effect persisted after adjustment for MFB and MUV (adjusted HR 0.25, 95%CI 0.12 to 0.52), suggesting that the diuretic in itself may exert a protective effect. This differs from the FACTT study, in which post-AKI furosemide use had a protective effect on 60-day mortality on univariate analysis, but not when adjusted for fluid balance [19]. The role of diuretics in AKI remains controversial. While three meta-analyses [34-36] have demonstrated a lack of association between diuretics and mortality, only two of the included studies enrolled ICU patients. Two observational studies in ICU settings showed conflicting results. The PICARD Study Group concluded that diuretic use increased the risk of death or non-recovery of renal function (OR 1.77, 95%CI 1.14 to 2.76) [37]. In contrast, the Beginning and Ending Supportive Therapy for the Kidney (BEST Kidney) investigators did not find a significant association between diuretic use and mortality (adjusted OR 1.21, 95%CI 0.96 to 1.5) [38]. Loop diuretics have effects that could be potentially beneficial in preventing or minimizing the severity of AKI. They reduce the oxygen demand and prevent hypoxic damage, and furosemide has been shown to improve renal hemodynamics, attenuate ischemic-related renal angiogenesis and reduce ischemic-induced apoptosis in animal models [39-41]. A pilot phase II randomized, blinded, placebo-controlled trial comparing furosemide to placebo in ICU patients with early AKI is in progress [42]; this study aims to compare the efficacy and safety of furosemide versus placebo on the progression of AKI severity and fluid balance. The results of this trial will help us to understand better the role of diuretics in AKI in the critical care setting.

Our study has important strengths including its multicenter design that contributes to reduce practice bias. The depth of data collection permitted the exploration of the effects of duration of oliguria and diuretic use, which confirmed the results of the main analysis. Our study also extends this continuous association of fluid balance, decreased urine volume and death to critically ill adults meeting the consensus definition for AKI (RIFLE). However, we recognize some limitations. Because of the observational nature of the study, we cannot establish a causal relationship between MFB, MUV and mortality. The modest sample size precluded extensive subgroup analysis and may have resulted in underestimation of the impact of known risk factors, such as SOFA score, on multivariable analysis. We did not collect data on actual diuretic dose, but the proportion of ICU days in which diuretics were used can be considered a reasonable surrogate. It is possible that the type of fluid (that is, colloid versus crystalloid, parenteral versus enteral), aside from the volume, influences outcomes. Unfortunately, this information was not available in our own study or in previous investigations on FO [9,13]. We believe this is an important issue that needs to be clarified with future studies. Nevertheless, our findings are consistent across the multiple sensitivity analyses performed and the association between fluid balance, urine volume and mortality appears robust.

## Conclusions

In summary, both fluid balance and urine volume were found to be independent predictors of mortality in adult critically ill patients with AKI. Of interest, diuretic use appeared to be independently associated with better survival in this study. The results of an ongoing phase II randomized controlled trial on diuretics in early AKI will help clarify this issue.

## Key messages

• In this multicenter observational study in ICU patients with AKI, non-survivors presented higher MFB (1.3 ± 1.2 versus 0.2 ± 0.7 L/day; *P *<0.001) and lower MUV (1.3 ± 0.9 versus 2.3 ± 1.0 L/day). Both higher fluid balance and a lower urine volume were shown to be independent predictors of 28-day mortality in this AKI population.

• The use of diuretics was associated with better survival.

## Abbreviations

AKI: acute kidney injury; ALI: acute lung injury; APACHE II: acute physiology and chronic health evaluation II; ARDS: acute respiratory distress syndrome; BEST Kidney: Beginning and Ending Supportive Therapy for the Kidney; FACTT: Fluid and Catheter Treatment Trail; FO: fluid overload; HR: hazard ratio; MFB: mean fluid balance; MUV: mean urine volume; NEFROINT: NEFROlogia e Cura INTensiva; OR: odds ratio; PICARD: Program to Improve Care in Acute Renal Disease; RIFLE: Risk of renal dysfunction, Injury to the kidney, Failure of kidney function, Loss of kidney function and End-stage kidney disease; RRT: renal replacement therapy; SAPS II: Simplified Acute Physiology Score II; SOAP: Sepsis Occurrence in Acutely Ill Patients; SOFA: Sequential Organ Failure Assessment; UO: urine output; VRWG: volume-related weight gain.

## Competing interests

The authors declare that they have no competing interests.

## Authors' contributions

The authors contributed as follows: Study conception and design - CT, FG, PP, DNC; patient enrollment and acquisition of data - NB, MI, SG, FF, MR, CBA, TB, MC, VM, PPelaia, DNC; data analysis and interpretation of the results - CT, NB, MI, DNC; Drafting of the manuscript - CT, DNC; critical revision of the manuscript - CT, FG, PP, NB, MI, SG, FF, MR, DNC; obtaining funding for the project - PP, CR. All authors have read and approved the final manuscript.

## Participating centers

St. Bortolo Hospital, Vicenza; University of Bari, Bari; Federico II University Hospital, Naples; University of Pisa, Pisa; University Hospital Umberto I - G.M. Lancisi - G. Salesi, Torrette, Ancona; Seconda Università degli Studi di Napoli, Naples; University of Rome "La Sapienza", Rome; Vita-Salute San Raffaele University, San Raffaele Scientific Institute, Milan; A.O.U.I. University of Verona, Verona; Catholic University of Sacred Heart, Rome

## Supplementary Material

Additional file 1 **Table S1**. Patient characteristics by presence or absence of AKI. Table S2: Patient characteristics by early or late AKI. Table S3: Sensitivity analysis using surrogates for mean urine volume and diuretic use. Variables considered for the final model included age, gender, co-morbid diseases, non-renal SOFA, sepsis, mean fluid balance, mean urine volume and diuretic use. For sensitivity analysis mean urine volume was replaced by either presence of oliguria or proportion of ICU days with oliguria as surrogates for mean urine volume; and diuretic use was substituted by proportion of ICU days receiving diuretics. AKI, acute kidney injury; SOFA, sequential organ failure assessment. Click here for file

## References

[B1] Lin SM Huang CD Lin HC Liu CY Wang CH Kuo HP A modified goal-directed protocol improves clinical outcomes in intensive care unit patients with septic shock: a randomized controlled trial Shock 2006 17 551 557 10.1097/01.shk.0000232271.09440.8f 17117128

[B2] Rivers E Nguyen B Havstad S Ressler J Muzzin A Knoblich B Peterson E Tomlanovich M Early goal-directed therapy in the treatment of severe sepsis and septic shock N Engl J Med 2001 17 1368 1377 10.1056/NEJMoa010307 11794169

[B3] Bagshaw SM Brophy PD Cruz D Ronco C Fluid balance as a biomarker: impact of fluid overload on outcome in critically ill patients with acute kidney injury Crit Care 2008 17 169 10.1186/cc6948 18671831 PMC2575565

[B4] Bagshaw SM Bellomo R Kellum JA Oliguria, volume overload, and loop diuretics Crit Care Med 2008 17 S172 178 10.1097/CCM.0b013e318168c92f 18382190

[B5] Mehta RL Bouchard J Controversies in acute kidney injury: effects of fluid overload on outcome Contrib Nephrol 2011 17 200 211 21921625 10.1159/000329410

[B6] Prowle JR Echeverri JE Ligabo EV Ronco C Bellomo R Fluid balance and acute kidney injury Nat Rev Nephrol 2010 17 107 115 10.1038/nrneph.2009.213 20027192

[B7] Uchino S Bellomo R Morimatsu H Sugihara M French C Stephens D Wendon J Honore P Mulder J Turner A Pulmonary artery catheter versus pulse contour analysis: a prospective epidemiological study Crit Care 2006 17 R174 10.1186/cc5126 17169160 PMC1794490

[B8] Sakr Y Vincent JL Reinhart K Groeneveld J Michalopoulos A Sprung CL Artigas A Ranieri VM High tidal volume and positive fluid balance are associated with worse outcome in acute lung injury Chest 2005 17 3098 3108 10.1378/chest.128.5.3098 16304249

[B9] Wiedemann HP Wheeler AP Bernard GR Thompson BT Hayden D deBoisblanc B Connors AF Jr Hite RD Harabin AL Comparison of two fluid-management strategies in acute lung injury N Engl J Med 2006 17 2564 2575 16714767 10.1056/NEJMoa062200

[B10] Brandstrup B Tonnesen H Beier-Holgersen R Hjortso E Ording H Lindorff-Larsen K Rasmussen MS Lanng C Wallin L Iversen LH Gramkow CS Okholm M Blemmer T Svendsen PE Rottensten HH Thage B Riis J Jeppesen IS Teilum D Christensen AM Graungaard B Pott F Danish Study Group on Perioperative Fluid Therapy Effects of intravenous fluid restriction on postoperative complications: comparison of two perioperative fluid regimens: a randomized assessor-blinded multicenter trial Ann Surg 2003 17 641 648 10.1097/01.sla.0000094387.50865.23 14578723 PMC1356139

[B11] Nisanevich V Felsenstein I Almogy G Weissman C Einav S Matot I Effect of intraoperative fluid management on outcome after intraabdominal surgery Anesthesiology 2005 17 25 32 10.1097/00000542-200507000-00008 15983453

[B12] Stein A de Souza LV Belettini CR Menegazzo WR Viegas JR Costa Pereira EM Eick R Araujo L Consolim-Colombo F Irigoyen MC Fluid overload and changes in serum creatinine after cardiac surgery: predictors of mortality and longer intensive care stay. A prospective cohort study Crit Care 2012 17 R99 10.1186/cc11368 22651844 PMC3580649

[B13] Vincent JL Sakr Y Sprung CL Ranieri VM Reinhart K Gerlach H Moreno R Carlet J Le Gall JR Payen D Sepsis in European intensive care units: results of the SOAP study Crit Care Med 2006 17 344 353 10.1097/01.CCM.0000194725.48928.3A 16424713

[B14] Goldstein SL Somers MJ Baum MA Symons JM Brophy PD Blowey D Bunchman TE Baker C Mottes T McAfee N Barnett J Morrison G Rogers K Fortenberry JD Pediatric patients with multi-organ dysfunction syndrome receiving continuous renal replacement therapy Kidney Int 2005 17 653 658 10.1111/j.1523-1755.2005.67121.x 15673313

[B15] Gillespie RS Seidel K Symons JM Effect of fluid overload and dose of replacement fluid on survival in hemofiltration Pediatr Nephrol 2004 17 1394 1399 10.1007/s00467-004-1655-1 15517417

[B16] Foland JA Fortenberry JD Warshaw BL Pettignano R Merritt RK Heard ML Rogers K Reid C Tanner AJ Easley KA Fluid overload before continuous hemofiltration and survival in critically ill children: a retrospective analysis Crit Care Med 2004 17 1771 1776 10.1097/01.CCM.0000132897.52737.49 15286557

[B17] Fulop T Pathak MB Schmidt DW Lengvarszky Z Juncos JP Lebrun CJ Brar H Juncos LA Volume-related weight gain and subsequent mortality in acute renal failure patients treated with continuous renal replacement therapy ASAIO J 2010 17 333 337 20559136 10.1097/MAT.0b013e3181de35e4 PMC2895683

[B18] Bouchard J Soroko SB Chertow GM Himmelfarb J Ikizler TA Paganini EP Mehta RL Fluid accumulation, survival and recovery of kidney function in critically ill patients with acute kidney injury Kidney Int 2009 17 422 427 10.1038/ki.2009.159 19436332

[B19] Grams ME Estrella MM Coresh J Brower RG Liu KD Fluid balance, diuretic use, and mortality in acute kidney injury Clin J Am Soc Nephrol 2011 17 966 973 10.2215/CJN.08781010 21393482 PMC3087792

[B20] Payen D de Pont AC Sakr Y Spies C Reinhart K Vincent JL A positive fluid balance is associated with a worse outcome in patients with acute renal failure Crit Care 2008 17 R74 10.1186/cc6916 18533029 PMC2481469

[B21] Van Biesen W Yegenaga I Vanholder R Verbeke F Hoste E Colardyn F Lameire N Relationship between fluid status and its management on acute renal failure (ARF) in intensive care unit (ICU) patients with sepsis: a prospective analysis J Nephrol 2005 17 54 60 15772923

[B22] Macedo E Malhotra R Bouchard J Wynn SK Mehta RL Oliguria is an early predictor of higher mortality in critically ill patients Kidney Int 2011 17 760 767 10.1038/ki.2011.150 21716258

[B23] Prowle JR Liu YL Licari E Bagshaw SM Egi M Haase M Haase-Fielitz A Kellum JA Cruz D Ronco C Tsutsui K Uchino S Bellomo R Oliguria as predictive biomarker of acute kidney injury in critically ill patients Crit Care 2011 17 R172 10.1186/cc10318 21771324 PMC3387614

[B24] Brivet FG Kleinknecht DJ Loirat P Landais PJ Acute renal failure in intensive care units--causes, outcome, and prognostic factors of hospital mortality; a prospective, multicenter study. French Study Group on Acute Renal Failure Crit Care Med 1996 17 192 198 10.1097/00003246-199602000-00003 8605788

[B25] Guerin C Girard R Selli JM Perdrix JP Ayzac L Initial versus delayed acute renal failure in the intensive care unit. A multicenter prospective epidemiological study. Rhone-Alpes Area Study Group on Acute Renal Failure Am J Respir Crit Care Med 2000 17 872 879 10.1164/ajrccm.161.3.9809066 10712336

[B26] Liano F Junco E Pascual J Madero R Verde E The spectrum of acute renal failure in the intensive care unit compared with that seen in other settings. The Madrid Acute Renal Failure Study Group Kidney Int Suppl 1998 17 S16 24 9580541

[B27] Rimmele T Kellum JA Oliguria and fluid overload Contrib Nephrol 2010 17 39 45 20427992 10.1159/000313719

[B28] Piccinni P Cruz DN Gramaticopolo S Garzotto F Dal Santo M Aneloni G Rocco M Alessandri E Giunta F Michetti V Iannuzzi M Belluomo Anello C Brienza N Carlini M Pelaia P Gabbanelli V Ronco C NEFROINT Investigators Prospective multicenter study on epidemiology of acute kidney injury in the ICU: a critical care nephrology Italian collaborative effort (NEFROINT) Minerva Anestesiol 2011 17 1072 1083 21597441

[B29] Garzotto F Piccinni P Cruz D Gramaticopolo S Dal Santo M Aneloni G Kim JC Rocco M Alessandri E Giunta F Michetti V Iannuzzi M Belluomo Anello C Brienza N Carlini M Pelaia P Gabbanelli V Ronco C NEFROINT investigation group RIFLE-based data collection/management system applied to a prospective cohort multicenter Italian study on the epidemiology of acute kidney injury in the intensive care unit Blood Purif 2011 17 159 171 10.1159/000322161 21228585

[B30] Knaus WA Draper EA Wagner DP Zimmerman JE APACHE II: a severity of disease classification system Crit Care Med 1985 17 818 829 10.1097/00003246-198510000-00009 3928249

[B31] Le Gall JR Lemeshow S Saulnier F A new Simplified Acute Physiology Score (SAPS II) based on a European/North American multicenter study JAMA 1993 17 2957 2963 10.1001/jama.1993.03510240069035 8254858

[B32] Vincent JL de Mendonca A Cantraine F Moreno R Takala J Suter PM Sprung CL Colardyn F Blecher S Use of the SOFA score to assess the incidence of organ dysfunction/failure in intensive care units: results of a multicenter, prospective study. Working group on "sepsis-related problems" of the European Society of Intensive Care Medicine Crit Care Med 1998 17 1793 1800 10.1097/00003246-199811000-00016 9824069

[B33] Bellomo R Ronco C Kellum JA Mehta RL Palevsky P Acute renal failure - definition, outcome measures, animal models, fluid therapy and information technology needs: the Second International Consensus Conference of the Acute Dialysis Quality Initiative (ADQI) Group Crit Care 2004 17 R204 212 10.1186/cc2872 15312219 PMC522841

[B34] Bagshaw SM Delaney A Haase M Ghali WA Bellomo R Loop diuretics in the management of acute renal failure: a systematic review and meta-analysis Crit Care Resusc 2007 17 60 68 17352669

[B35] Ho KM Sheridan DJ Meta-analysis of frusemide to prevent or treat acute renal failure BMJ 2006 17 420 10.1136/bmj.38902.605347.7C 16861256 PMC1553510

[B36] Zacharias M Conlon NP Herbison GP Sivalingam P Walker RJ Hovhannisyan K Interventions for protecting renal function in the perioperative period Cochrane Database Syst Rev 2008 4 CD003590 18843647 10.1002/14651858.CD003590.pub3

[B37] Mehta RL Pascual MT Soroko S Chertow GM Diuretics, mortality, and nonrecovery of renal function in acute renal failure JAMA 2002 17 2547 2553 10.1001/jama.288.20.2547 12444861

[B38] Uchino S Doig GS Bellomo R Morimatsu H Morgera S Schetz M Tan I Bouman C Nacedo E Gibney N Tolwani A Ronco C Kellum JA Beginning and Ending Supportive Therapy for the Kidney (B.E.S.T. Kidney) Investigators Diuretics and mortality in acute renal failure Crit Care Med 2004 17 1669 1677 10.1097/01.CCM.0000132892.51063.2F 15286542

[B39] Aravindan N Aravindan S Riedel BJ Weng HR Shaw AD Furosemide prevents apoptosis and associated gene expression in a rat model of surgical ischemic acute renal failure Ren Fail 2007 17 399 407 10.1080/08860220701263671 17497460

[B40] Aravindan N Shaw A Effect of furosemide infusion on renal hemodynamics and angiogenesis gene expression in acute renal ischemia/reperfusion Ren Fail 2006 17 25 35 10.1080/08860220500461229 16526316

[B41] Heyman SN Rosen S Epstein FH Spokes K Brezis ML Loop diuretics reduce hypoxic damage to proximal tubules of the isolated perfused rat kidney Kidney Int 1994 17 981 985 10.1038/ki.1994.132 8007601

[B42] Bagshaw SM Gibney RT McAlister FA Bellomo R The SPARK Study: a phase II randomized blinded controlled trial of the effect of furosemide in critically ill patients with early acute kidney injury Trials 2010 17 50 10.1186/1745-6215-11-50 20459801 PMC2874544

