# Supplementary material for: Fluid balance and urine volume are independent predictors of mortality in acute kidney injury
Source: Crit Care. 2013 Jan 24;17(1):R14. doi: 10.1186/cc12484 (PMC4057508; doi:10.1186/cc12484)
Supplement: Additional file 1 — Table S1. Patient characteristics by presence or absence of AKI. Table S2: Patient characteristics by early or late AKI. Table S3: Sensitivity analysis using surrogates for mean urine volume and diuretic use. Variables considered for the final model included age, gender, co-morbid diseases, non-renal SOFA, sepsis, mean fluid balance, mean urine volume and diuretic use. For sensitivity analysis mean urine volume was replaced by either presence of oliguria or proportion of ICU days with oliguria as surrogates for mean urine volume; and diuretic use was substituted by proportion of ICU days receiving diuretics. AKI, acute kidney injury; SOFA, sequential organ failure assessment. [file cc12484-S1.DOC]

**Additional file 1**

**Title: Additional analysis of patient characteristics and sensitivity analysis**

Description: Additional file 1 document contains additional information relevant for the interpretation of the manuscript. In this section we present:

-Table S1 which shows patient characteristics by presence or absence of AKI;

-Table S2 demonstrates AKI patient characteristics according to timing of AKI development;

- Table S3 that shows the results of the sensitivity analysis performed where mean urine volume was replaced by either presence of oliguria or proportion of ICU days with oliguria as surrogates for mean urine volume; and diuretic use was substituted by proportion of ICU days receiving diuretics.

Table S1: Patient characteristics by presence or absence of AKI

|  | **All (n=573)** | **AKI (n=132)** | **Non-AKI (n=441)** | **P** |
| --- | --- | --- | --- | --- |
| Male gender | 341 (59.5%) | 84 (63.6%) | 257 (58.3%) | 0.312 |
| Age | 63.0 ± 17.3 | 66.3 ± 14.1 | 62.0 ± 18.0 | 0.012 |
| **ICU Admission** | | | | |
| SAPS II | 45.3 ± 14.6 | 50.0 ± 14.5 | 43.91 ± 14.3 | <0.001 |
| SOFA | 5.7 ± 3.6 | 8.0 ± 4.1 | 5 ± 3.1 | <0.001 |
| Non-renal SOFA | 5.1 ± 3.2 | 6.6 ± 3.5 | 4.7 ± 2.9 | <0.001 |
| APACHE II | 18.8 ± 7.6 | 22.3 ± 8.4 | 17.7 ± 7.0 | <0.001 |
| Vasoactive therapy | 148 (25.8%) | 56 (42.4%) | 92 (20.9%) | <0.001 |
| Mechanical ventilation | 419 (73.1%) | 96 (72.7%) | 323 (73.2%) | 0.911 |
| Serum creatinine (mg/dL) | 1.31 ± 1.20 | 2.35 ± 2.01 | 1.00 ± 0.49 | <0.001 |
| **Comorbid diseases** | | | | |
| Diabetes | 108 (18.8%) | 41 (31.1%) | 67 (15.2%) | <0.001 |
| Cardiovascular | 249 (43.5%) | 66 (50%) | 183 (41.5%) | 0.089 |
| Hypertension | 269 (46.9%) | 67 (50.8%) | 202 (45.8%) | 0.322 |
| Chronic kidney disease | 36 (6.3%) | 19 (14.4%) | 17 (3.9%) | <0.001 |
| **Category of ICU admission diagnosis** | | | | |
| Respiratory | 158 (27.6%) | 44 (33.3%) | 114 (25.9%) | 0.097 |
| Neurologic | 97 (16.9%) | 15 (11.4%) | 82 (18.6%) | 0.063 |
| Trauma | 82 (14.3%) | 8 (6.1%) | 74 (16.8%) | 0.002 |
| Cardiovascular | 70 (12.2%) | 25 (18.9%) | 45 (10.2%) | 0.010 |
| Sepsis | 32 (5.6%) | 15 (11.4%) | 17 (3.9%) | 0.002 |
| **ICU course** | | | | |
| Sepsis | 149 (26%) | 61 (46.2%) | 88 (20%) | <0.001 |
| Oliguria | 91 (15.9%) | 91 (68.9%) | N/A | N/A |
| Use of diuretics (%) | 365 (63.7%) | 102 (77.3%) | 263 (59.6%) | <0.001 |
| **Outcomes** | | | | |
| Length of ICU stay (days) | 10.1 ± 12.0 | 15.3 ± 16.3 | 8.6 ± 9.8 | <0.001 |
| ICU mortality | 125 (21.8%) | 66 (50.0%) | 59 (13.4%) | <0.001 |

Acute kidney injury (AKI) was defined by a renal SOFA score≥3. ICU, intensive care unit; SAPS II, Simplified Acute Physiology Score II; SOFA, Sequential Organ Failure Assessment; APACHE II, Acute Physiology and Chronic Health Evaluation II.

Table S2: Patient characteristics by early or late AKI

|  | **Early AKI (n= 83)** | **Late AKI (n=49)** | **P** |
| --- | --- | --- | --- |
| Male gender | 49 (59%) | 35 (71.4%) | 0.191 |
| Age | 66.6 ± 14.3 | 65.7 ± 13.8 | 0.741 |
| **ICU Admission** | | | |
| SAPS II | 52.1 ± 15.1 | 46.5 ± 12.8 | 0.029 |
| SOFA score | 8.9 ± 4.5 | 6.6 ± 2.8 | 0.02 |
| Non-renal SOFA | 7.0 ± 3.8 | 5.8 ± 2.7 | 0.050 |
| APACHE II | 23.8 ± 8.7 | 19.9 ± 7.1 | 0.009 |
| Vasoactive therapy | 41 (49.4%) | 15 (30.6%) | 0.026 |
| Mechanical ventilation | 61 (73.5%) | 35 (71.4%) | 0.475 |
| Serum creatinine (mg/dL) | 2.91 ± 2.28 | 1.39 ± 0.83 | <0.001 |
| **ICU Course** | | | |
| Mean Fluid Balance/24h (L) | 0.84 ± 1.34 | 0.58 ± 0.74 | 0.217 |
| Oliguria | 63 (75.9%) | 28 (57.1%) | 0.032 |
| Duration of oliguria (days) (% ICU days) | 3.0 ± 6.5  (32.3 ± 34.8%) | 2.3 ± 4.7  (11.5 ± 15.6%) | 0.503  (<0.001) |
| Mean Urine Volume/24h (L) | 1.56 ± 1.09 | 2.24 ± 0.92 | <0.001 |
| Diuretic use | 63 (75.9%) | 39 (79.6%) | 0.673 |
| Duration of diuretic use (days) (% ICU days) | 6.2 ± 8.2  (51.1 ± 38.3%) | 11.9 ± 14.6  (51.0 ± 36.9%) | 0.004  (0.987) |
| **Outcome** | | | |
| RRT (%) | 40 (48.2%) | 27 (55.1%) | 0.475 |
| Duration of RRT (days) (%ICU days) | 3.3 ± 7.9  (29.4 ± 36.0%) | 3.1 ± 5.0  (13.0 ± 16.8%) | 0.891  (0.003) |
| RRT free days | 7.8 ± 8.2 | 19.4 ± 18.0 | <0.001 |
| Length of ICU stay (days) | 11.1 ± 11.8 | 22.5 ± 20.1 | <0.001 |
| ICU mortality | 39 (47.0%) | 27 (55.1%) | 0.471 |

AKI, acute kidney injury; ICU, intensive care unit; SAPS II, Simplified Acute Physiology Score II; SOFA, Sequential Organ Failure Assessment; APACHE II, Acute Physiology and Chronic Health Evaluation II; RRT, renal replacement therapy.

Table S3: Sensitivity analysis using surrogates for mean urine volume and diuretic use

|  | **Adjusted Hazard Ratio (95% CI)** | | | | | |
| --- | --- | --- | --- | --- | --- | --- |
|  | **Final model** | **Alternative models** | | | | |
|  |  | **Model A** | **Model B** | **Model C** | **Model D** | **Model E** |
| **Mean Fluid Balance/24h (L)** | 1.67  (1.33-2.09) | 1.86  (1.50-2.32) | 1.72  (1.37-2.15) | 1.73  (1.38-2.18) | 1.98  (1.59-2.48) | 1.81  (1.43-2.29) |
| **Mean Urine Volume/24h (L)** | 0.47  (0.33-0.67) | - | - | 0.53  (0.36-0.76) | - | - |
| **Oliguria** | - | 1.35  (0.67-2.7) | - | - | 1.04  (0.51-2.12) | - |
| **% ICU days with oliguria** | - | - | 1.01  (1.003-1.02) | - | - | 1.01  (1.00-1.02) |
| **Diuretics** | 0.25  (0.12-0.52) | 0.25  (0.12-0.50) | 0.24  (0.12-0.50) | - | - | - |
| **% ICU days receiving diuretics** | - | - | - | 0.99  (0.98-0.996) | 0.98  (0.97-0.99) | 0.98  (0.97-0.99) |

Variables considered for the final model included age, gender, co-morbid diseases, non-renal SOFA, sepsis, mean fluid balance, mean urine volume and diuretic use. For sensitivity analysis mean urine volume was replaced by either presence of oliguria or proportion of ICU days with oliguria as surrogates for mean urine volume; and diuretic use was substituted by proportion of ICU days receiving diuretics. CI, confidence interval; ICU, intensive care unit.
